# Performance of Different Crop Models in Simulating Soil Temperature

**DOI:** 10.3390/s23062891

**Published:** 2023-03-07

**Authors:** Janani Kandasamy, Yuan Xue, Paul Houser, Viviana Maggioni

**Affiliations:** 1 Sid and Reva Dewberry Department of Civil, Environmental, and Infrastructure Engineering, George Mason University, Fairfax, VA 22042, USA; 2 Department of Geography and Geoinformation Science, George Mason University, Fairfax, VA 22042, USA

**Keywords:** soil temperature, land surface modeling, crop model, reconstructed mean, Central Valley California

## Abstract

Soil temperature is one of the key factors to be considered in precision agriculture to increase crop production. This study is designed to compare the effectiveness of a land surface model (Noah Multiparameterization (Noah-MP)) against a traditional crop model (Environmental Policy Integrated Climate Model (EPIC)) in estimating soil temperature. A sets of soil temperature estimates, including three different EPIC simulations (i.e., using different parameterizations) and a Noah-MP simulations, is compared to ground-based measurements from across the Central Valley in California, USA, during 2000–2019. The main conclusion is that relying only on one set of model estimates may not be optimal. Furthermore, by combining different model simulations, i.e., by taking the mean of two model simulations to reconstruct a new set of soil temperature estimates, it is possible to improve the performance of the single model in terms of different statistical metrics against the reference ground observations. Containing ratio (CR), Euclidean distance (dist), and correlation co-efficient (R) calculated for the reconstructed mean improved by 52%, 58%, and 10%, respectively, compared to both model estimates. Thus, the reconstructed mean estimates are shown to be more capable of capturing soil temperature variations under different soil characteristics and across different geographical conditions when compared to the parent model simulations.

## 1. Introduction

Terrestrial water is crucial for healthy ecosystems, energy, and food production, socio-economic development, and human survival [1]. The depletion of water resources endangers food security and affects the well-being of the humankind globally [2,3,4,5]. Food demand is facing many challenges, including but not limited to climate variability, water scarcity, variation in soil fertility, environmental pollution, and change in vegetation pattern [6]. Food demand is expected to be more than 80–100% of the current demand by 2050 [1,7,8].

In agriculture, being the largest water consumption sector, the primary focus to reduce water scarcity has been on advancing the efficiency of water supply systems and irrigation water use [5,9,10]. Precision agriculture (PA) is a key technological solution to tackle food and water scarcity [11]. PA is “a holistic, sustainable, innovative systems approach that assists farmers in production management” [12]. It is a modern approach based on farm and irrigation management to improve the efficiency of agricultural resources, thereby maximizing the crop productivity and yield through technologies that identify, analyze, and monitor variability within a field and optimize profitability, sustainability, and protection of the land resources [13,14,15]. Precision farming has the potential to provide economic and environmental benefits by reducing the use of water, fertilizers, and pesticides, in addition to farm equipment, by applying the right amount of input (water, fertilizer, herbicides, etc.) at the right location and time [16,17].

Various parameters are involved in plant growth, including water level, soil nutrients, temperature, and others [18]. Soil temperature is one of the principal factors to be considered in irrigating agricultural fields, as it influences the geochemical and physiochemical processes, nutrient release, microbial activity, and organic matter decomposition under the ground surface [19,20]. High soil temperatures may impact crop stress and reduce crop yield [21]. For most crops, the optimum soil temperature range is between 20 °C and 30 °C, and plant growth declines if temperature is outside such bounds [19]. Soil temperature is also influenced by soil characteristics and meteorological variables, such as air temperature and solar radiation, which affect plant development, maturation, and a series of biological and physicochemical soil processes [22,23,24,25].

Over the years, numerous field-scale crop models (e.g., Environmental policy Integrated Climate (EPIC)) and continental-scale land surface models (e.g., Noah Multiparameterization (Noah-MP)) have been proposed, each with its strengths and weaknesses. All these models have the capacity to predict a variety of hydrological variables at different scales, including soil temperature, but none is able to consistently outperform the others under heterogeneous climatologies and terrains [26,27,28,29]. There is no perfect model because all models are subjective to model errors arising from imperfect model parameterizations, as well as errors in the boundary conditions. Nevertheless, previous studies have showed how more robust, reliable, and efficient estimates can be obtained by combining multiple model simulations [30,31,32,33]. In this study, the crop model EPIC and land surface model Noah-MP are used for soil temperature simulation. Although both Noah-MP and EPIC have been widely evaluated separately at different locations [25,34,35,36,37,38,39], to our knowledge, a side-by-side comparison of their performance has not been done yet, let alone the impact of combining the two models (different in nature) to produce a new estimate of soil temperature. Table 1 lists the literature reviewed about the simulation process and performance of EPIC and Noah-MP.

Both EPIC and Noah-MP are widely used process-based models. We choose not to use machine learning based algorithms for predicting soil temperature (or other hydrological variables) because most of these models are considered “black box” [47,48,49,50]. We believe it is important to explain the model behavior based on sound physics. Furthermore, the California drought and other recent anomalies alert us that we are likely entering a future of heightened climatological extremes: droughts, heat waves, etc. Under such nonstationary conditions, these events may fail outside a machine learning training dataset, and therefore yield sub-optimal estimates.

The main goal of this work is to estimate soil temperature using different models and compare the performance of each model simulations against ground-based measurements. In this study, soil temperature is estimated using both EPIC (and variations in its parameterization) and Noah-MP to compare the performance of each model simulation against ground-based measurements. Three different schemes to calculate soil temperature are used in EPIC (for a total of three different outputs) and a new set of soil temperature estimates is also proposed by merging EPIC and Noah-MP through an arithmetic mean. Statistical metrics are then calculated to quantitatively evaluate the performance of individual model simulations along with the newly merged estimates. The manuscript is organized as follows. Section 2 presents the study domain, data, and methodology used to estimate soil temperature and the statistical metrics used to evaluate different model simulations. Section 3 summarizes and analyzes the results. Section 4 discusses the major findings. Conclusions and future research directions are presented in Section 5.

## 2. Methodology

### 2.1. Study Sites 

**Figure 1:**
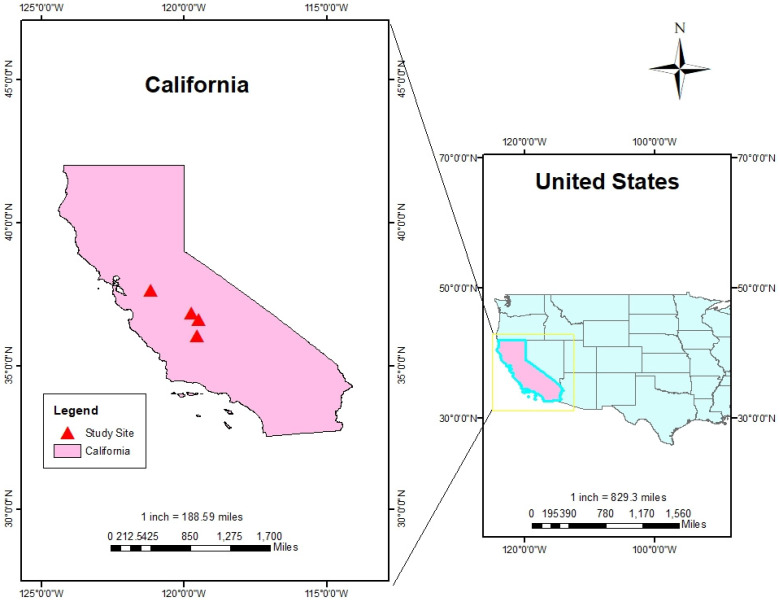
Location of study sites in California.

The study focuses on four sites (see Figure 1) located in the Californian Central Valley, also known as the Great Valley. The Central Valley is a broad, elongated, flat valley that covers about 20,000 square miles, which corresponds to about 11% of California’s total land area. The diverse climates across California (highland, cool interior, desert, Mediterranean) allow for a wide range of agricultural goods to be grown throughout the state (more than 250 different crops). It is California’s most productive agricultural region and produces 25% of the Nation’s food, including 40% of the Nation’s vegetables, fruits, nuts, and other table foods. Historical droughts that affected this region resulted in severe economic and environmental impacts, which posed a critical challenge to the food production system [51,52,53,54,55,56].

Four sites (Fresno, Modesto, Parlier, and Corcoran) were selected to evaluate the performance of different models in simulating soil temperature. These sites are in four different counties across the Central Valley and characterized by different types of crops and local conditions. Specifically, the Modesto site is located at a slightly higher elevation than the others and local activities include herding and agriculture (mainly fruits and nuts). The Parlier station is characterized by large fields of cotton, grapes, and orchards. The Fresno site is only used for livestock in small paddock with large pasture settings. The Corcoran site is adjacent to extensive cotton fields. Since the daily meteorological data record are incomplete, this site is used to evaluate the reliability of the EPIC simulations. Please refer to Table 2 for a summary of local characteristics of the four sites.

### 2.2. Experimental Set-Up

#### 2.2.1. Noah-MP

The Noah-MP model is integrated forward in time at a time step of 15 min from 1 January 1990 to 1 January 2020 on a 10 km spatial grid using the NASA Land Information System (LIS) version 7.4.5-557WW downloaded from GitHub (https://github.com/NASA-LIS/LISF; [57]). Noah-MP outputs are generated on a daily-averaged time step. The model is spun up, reaching quasi-equilibrium by looping one time through the period from 1 January 1990 to 1 January 2020. 

We run all Noah-MP simulations within the NASA Land Information System, where a simplified representation of the crop phenology, irrigation scheduling, along with a groundwater abstraction scheme can be specified. Follow-up studies should investigate other land surface models and include irrigation schemes to analyze their performance during irrigation seasons.

Noah-MP derived soil temperature estimates are forced by Modern-Era Retrospective analysis for Research and Applications, Version 2 (MERRA-2; [58]) forcing fields, which include air temperature, specific humidity, downward longwave flux, downward shortwave flux, zonal wind, meridional wind, surface pressure, and total corrected precipitation. We choose total corrected precipitation because it yields better performance compared to that using the uncorrected precipitation field (results not shown). It is also important to note that additional physically based downscaling procedure (i.e., temperature or humidity lapse rate corrections; [59,60]) is applied to the atmospheric forcing variables in this study. 

A four-layer soil column configuration is used in the Noah-MP model. The thickness of each soil layer (from top to bottom) is 10 cm, 30 cm, 60 cm, and 100 cm, respectively. Using the ground heat flux (at the surface) as the upper boundary, the soil temperatures of the four-layer soil column are solved together through a tri-diagonal matrix of the implicit time scheme with soil thermal diffusivity properties [45,61]. Soil temperature values obtained from Noah-MP represent the temperatures at each soil layer center (from the ground surface) at 5 cm (layer #1), 25 cm (layer #2), 70 cm (layer #3), and 150 cm (layer #4), respectively. Therefore, the soil temperature at 15 cm is calculated as the mean of temperature between layer #1 and layer #2. 

#### 2.2.2. EPIC

Originally called as the Erosion Productivity Impact Calculator, EPIC was developed by the United States Department of Agriculture (USDA) in the early 1980s [62] and later renamed as the Environmental Policy Integrated Climate model. It is a soil and crop model initially formulated to calculate the effect of soil erosion on crop productivity. Since then, the model has been actively maintained and expanded to improve the simulation of plant growth by including various physical and biochemical processes. The nine different components of the model are erosion, weather, hydrology, nutrients, plant environmental control, plant growth, soil temperature, tillage, and economic budgets. EPIC is a field scale model. It runs continuously on a daily time step and can simulate up to ten layers of soil profile for a hundred or thousands of years (i.e., long-term simulation). 

Numerous studies have been conducted using different components of EPIC in the U.S. and in other parts of the world. Example applications include climate change and atmospheric CO_2_ impact on crop productivity [44,63]; irrigation scheduling [39]; soil temperature [25]; crop yield and sensitivity [40], and wind erosion sediment loss [37].

**Figure 2:**
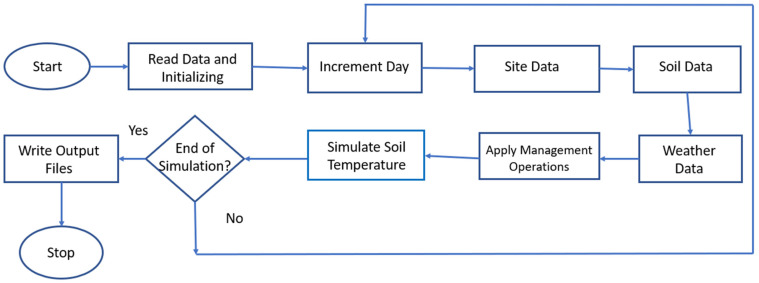
Key Process in EPIC1102 File Structure.

Epic version 1102 is used for all EPIC derived simulations in this study. We run EPIC at the four sites one at a time individually. The key process in the file structure of EPIC1102 is shown in Figure 2. The input data includes characteristics about soil, site (topography and geography data), and weather. For consistency with Noah-MP, the soil column is divided into four layers at the depth of 10 cm, 30 cm, 60 cm, and 100 cm. Daily weather information and monthly weather statistics were generated using the Weather Import and WXPM V3020 program available on the Texas A&M AgriLife website (https://epicapex.tamu.edu/software/weather-import accessed on 4 April 2022). Daily weather data are obtained from the California Irrigation Management Information System (CIMIS). CIMIS is a program unit in the California Department of Water Resources with 145 automated weather stations placed throughout California, mainly developed for farmers/irrigators for water resources management (https://cimis.water.ca.gov/Default.aspx accessed on 10 March 2022). Daily soil temperature is measured at a depth of 15 cm below ground with a soil thermistor. The accuracy of the sensor is within ±0.4 °C. Throughout the evaluation period between 2000 and 2019 across all sites, no frozen soil conditions (soil temperature < 273.15 K) were encountered, based on the CIMIS in-situ measurements.

The EPIC simulation run for 30 years from 1990 to 2019. The first 10 years in the time series are used as model spin up, and therefore results are not included in the evaluation phase. Soil temperature estimates are simulated using three different submodels: the original cosine (EPIC-original), the enhanced cosine (EPIC-enhanced), and the pseudo heat transfer (EPIC-pseudo). Each submodel considers different set of factors to estimate soil temperature. Specifically, the EPIC-original approach considers air temperature and solar radiation only. The EPIC-enhanced considers soil cover factors (e.g., snow, plant, and litter) in addition to solar radiation and air temperature. The EPIC-pseudo includes an extra module simulating heat transfer between different layers along with all other factors mentioned above. To be consistent with the Noah-MP soil layering profile, in each EPIC-based approach, the soil temperature was predicted at four layers, and soil temperature at 15 cm depth was calculated and evaluated in this study. The three options to estimate soil temperature were selected one at a time in the EPIC control file. Only the key parameterizations are described in this study for clarity. Please refer to Doro et al. (2021) [25] for detailed formulations. 

Built on top of EPIC-original, EPIC-enhanced considers the effect of soil cover factors: snow cover, total above ground plant material cover, and the fraction of ground covered by the leaf area index, on the soil surface temperature. These three parameterizations are computed as follows:

(1) $$fc_{snow}=\min\left(P87,\frac{Z_{snow}}{Z_{snow}+\;e^{\left(1.44-0.37*Z_{snow}\right)}}\right)$$

(2) $$fc_{biom}\;=\min\left(P95,\max\left(fc_{plant},\frac{fc_{rsd}}{fc_{rsd}+e^{\left(0.12-0.12*fc_{rsd}\right)}}\right)\right)$$

(3) $$fc_{plant}=\frac{LAI}{LAI+e^{\left(1.75-1.75*LAI\right)}}$$

where *fc_snow_* is the snow cover factor, P87 is a parameter for setting an upper limit on the snow cover factor, *Z_snow_* is the actual snow cover, *fc_biom_* is the plant material cover factor, P95 is a parameter for setting an upper limit on the vegetative cover factor, *fc_plant_* is the fraction of soil surface covered by plants, *fc_rsd_* is the fraction of soil surface covered by plant residue, and LAI is the leaf area index. In the EPIC-pseudo approach, the heat transfer efficiency between layers is considered for soil temperature estimation. For each soil layer, the transfer coefficient U is calculated as follows:

(4) $$U=\;0.6\;\times \;\mathrm{fbd}\;\times \;\mathrm{fsw}\;\times \;(1-{\mathrm{fc}}_{\mathrm{snow}})$$

where fbd is the bulk density factor and fsw is the soil water factor.

### 2.3. Model Evaluation

The soil temperatures simulated from three different approaches provided by EPIC are compared with Noah-MP model at four sites in Central Valley of California: Corcoran, Fresno, Modesto, and Parlier. Each set of model simulations is then evaluated against observed soil temperatures at 15 cm. In addition to the estimates derived by EPIC or Noah-MP, we also reconstructed a new set of estimates by taking the mean of EPIC-Pseudo and Noah-MP (Reconstruct-mean hereinafter), which has shown to produce the best performance (as demonstrated in Section 3). Although we reconstructed the mean using different combinations of the model estimates, the mean of EPIC-Pseudo and Noah-MP produced the best performance. Therefore, results for the other trials are not presented in this manuscript. 

The overlapping simulation period between 01 January 2000 and 31 December 2019 is selected as the evaluation period. Note that, in this study, we only focus on the time with minimized irrigation activities and leave the evaluations during the active irrigated seasons for future work.

In the model evaluation phase, we consider a set of three goodness-of-fit statistics: containing ratio, Euclidean distance, and correlation coefficient. The containing ratio (CR) is computed as:

(5) $$CR\left(\%\right)=\;\frac{\sum_{i=1}^{N}I\left[O\left(x_{est,i}\right)\right]}{N}*100$$

where *I*[*O*(*x_est,i_*)]=1 if *x_meas,min,i_*≤ *x_est,i_* ≤ *x_meas,max,i_*. *x_est,I_* denotes model estimates at time *i*, *x_meas,min,i_* denotes the minimum value of measurements at time *I*, and *x_meas,max,i_* denotes the maximum value of measurement at time *i*. *N* denotes the total number of days. Therefore, a higher CR means model estimates hit in between the minimum and maximum enclosed area more, which yields a better model performance. The Euclidean distance (dist) is computed as:

(6) $$dist\;\left(K\right)=\;\sqrt{\overset{N}{\underset{i=1}{\sum }}{\left(x_{est,i}-\;x_{meas,center,i}\right)}^{2}}$$

where *x_meas,center,i_* denotes the centered measurement, i.e., mean of minimum and maximum, value at time *i*. Therefore, a smaller dist value implies shorter distance to the measurements’ centerline, which results in better model performance. The correlation coefficient (*R*; [64]) is computed as:

(7) $$R\left(-\right)=\;\frac{\sum_{i=1}^{N}\left(x_{est,i}-\;\bar{x_{est}}\right)\;\left(x_{meas,center,i}-\;\bar{x_{meas,center}}\right)}{\sqrt{\sum_{i=1}^{N}{\left(x_{est,i}-\;\bar{x_{est}}\right)}^{2}\;\sum_{i=0}^{N}{\left(x_{meas,center,i}-\;\bar{x_{meas,center}}\right)}^{2}}}$$

where $\bar{x_{est}}$ denotes the time-averaged model estimates, and $\bar{x_{meas,center}}$ denotes the time-averaged centered measurements. A higher *R* demonstrates better correlations with the reference. Overall, a relatively high CR, or small dist, or high *R* is deemed as a higher level of accuracy in the model estimates. Through these goodness-of-fit statistics, we can evaluate different model derived estimates and have a quantitative understanding of the model performance.

## 3. Results

Daily average soil temperature estimated by the two models (EPIC and Noah-MP) are evaluated by comparing against CIMIS-measured daily minimum and maximum soil temperature at 15 cm soil depth from 2000 to 2020. In general, it is difficult to conclude whether EPIC alone or Noah-MP alone is consistently superior to the other across all selected study sites in terms of all goodness-of-fit statistics. That is, at some sites or at certain times, Noah-MP performs better, whereas at other sites or other periods, EPIC performs better.

**Figure 3:**
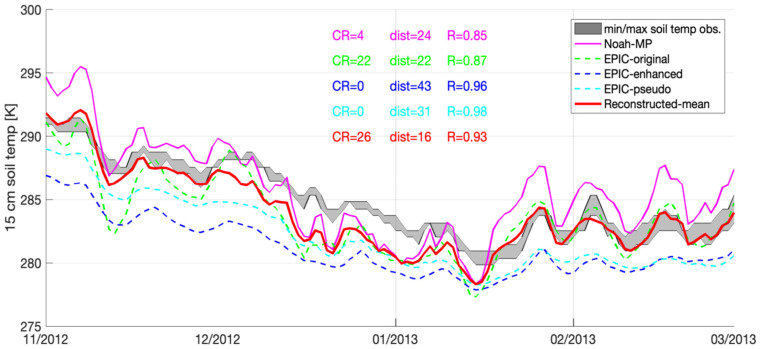
Time series of soil temperature at 15 cm depth obtained from Noah-MP, EPIC-original, EPIC-Enhanced, EPIC-pseudo, as well as ground-based CIMIS measurements at CIMIS#80 (Fresno) from November 2012 to March 2013.

According to the average statistics summarized in Table 3, Table 4, Table 5 and Table 6, among all the EPIC simulated results, EPIC-original yields the best performance in terms of CR and dist at the Fresno, Modesto, and Corcoran sites. However, it yields the poor performance compared to other two approaches in terms of R at all four selected sites. This is because at Fresno, Modesto, and Corcoran sites, both EPIC-Pseudo and EPIC-enhanced yield consistent negative biases, whereas EPIC-original often yields a relatively high temporal variations in the soil temperature estimates. An example is shown in Figure 3 at the Fresno site between November 2012 and March 2013. Both EPIC-pseudo and EPIC-enhanced never hit the CIMIS-enclosed min/max area because of their systematic negative biases, and therefore both yield the lowest CR (=0), and worst dist values (i.e., greater than 31). The relatively high temporal variations in the EPIC-original simulated soil temperature are beneficial for improving the model performance in terms of CR and dist. However, it significantly degrades the model performance in terms of R. This is likely due to the lack of constraint in soil cover factors, such as seasonal snow cover, embedded within the EPIC-original approach [25].

**Figure 4:**
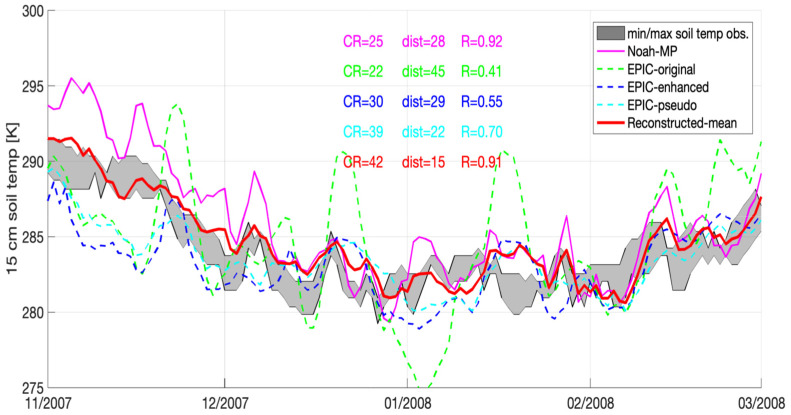
Time series of soil temperature at 15 cm depth obtained from Noah-MP, EPIC-original, EPIC-Enhanced, EPIC-pseudo, as well as ground-based CIMIS measurements at CIMIS#39 (Parlier) from November 2007 to March 2008.

The high temporal variation shown by the EPIC-original does not always guarantee the best results. An example is presented in Figure 4 at the Parlier station between November 2007 and March 2008. Figure 4 shows that EPIC-original yields the worst performance in terms of CR (=22), dist (=45), and R (=0.41). On the other hand, EPIC-pseudo yields the best performance in terms of CR (=39), and dist (=22). Although compared to EPIC-original, both EPIC-pseudo and EPIC-enhanced improve R to some extent. However, R values range between 0.55 and 0.70, which still indicate mildly weak correlations. The relatively poor performance in R witnessed in all EPIC results may be due to uncertainties associated with model inputs (e.g., soil properties) and model structure (e.g., simplifying assumptions in mathematical terms to represent complex soil-plant-atmosphere systems; [25]). In addition, system-defaulted parameterizations (see Section 2.2 for details) used in both EPIC-enhanced and EPIC-pseudo approaches may yield suboptimal estimates, which suggests that when relying on EPIC-alone simulations, site-specific calibrations of these parameters in both EPIC-enhanced and EPIC-pseudo can be crucial.

**Figure 5:**
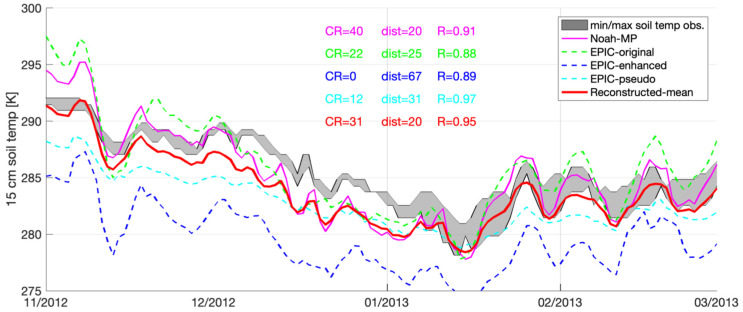
Time series of soil temperature at 15 cm depth obtained from Noah-MP, EPIC-original, EPIC-Enhanced, EPIC-pseudo, as well as ground-based CIMIS measurements at CIMIS#20 (Corcoran) from November 2012 to March 2013.

Compared with all EPIC model performance, Noah-MP yields slightly better performance especially in terms of dist (see Table 3, Table 4, Table 5 and Table 6). For example, except for the Modesto station, compared to the EPIC simulations, Noah-MP yields the lowest value in dist across the other stations. In terms of CR, Noah-MP yields decent performance at both Corcoran and Parlier sites. At the Corcoran site, part of the in-situ daily meteorological data is missing (i.e., nearly 38% of the air temperature and 46% of solar radiation data). EPIC relies heavily on daily in-situ meteorological data (e.g., daily minimum and maximum solar radiation) and when missing an input, EPIC tabulates daily values using system-defaulted long-term monthly means and standard deviations. Under such a scenario, EPIC-derived results are not reliable, since they depart from the ground-based measurements (e.g., dist ranges from 31.7 to 69.0). On the other hand, Noah-MP simulations driven by the MERRA-2 reanalysis product, which do not rely on in-situ data, yield the best performance in terms of CR and dist when compared to all EPIC estimates. The reconstructed mean estimates do not improve the original simulations in terms of dist (Table 3, Table 4 and Table 5) and a slight degradation is witnessed in CR compared to Noah-MP. This suggests that averaging EPIC and Noah-MP estimates may not be ideal when EPIC input data are not reliable. Figure 5 shows an example of the soil temperature evaluation at the Corcoran site. Estimated soil temperature at the Corcoran site shows variations in peak and low points, as shown in Figure 5. This is possibly due to the variation of air temperature because both are determined by the energy balance at the ground surface.

Among all four selected stations, the average R values obtained from Noah-MP ranging from 0.83 to 0.87, which is more stable and less variable than any of EPIC derived estimates ranging from 0.39 to 0.94. The relatively performance obtained in the R statistics from Noah-MP might be because the local terrain is relatively flat, and therefore, model estimates driven by MERRA-2 at 10 km may be able to represent local conditions on average. However, we do acknowledge that Noah-MP estimates at 10 km are too coarse, and they may not be able to fully capture local heterogeneity, as shown in Figure 3 at the Fresno site as an example. That is, although Noah-MP yields a much better performance in terms of dist relative to EPIC-enhanced and EPIC-pseudo, Noah-MP’s performance in R is much worse.

Therefore, to generate more reliable and stable soil temperature estimates, we derive a new set of estimates by fusing Noah-MP and EPIC simulations. Specifically, we compute the new reconstructed mean time series by taking the mean of the Noah-MP and EPIC-pseudo derived estimates. This, in general, yields the best and most stable performance, especially in terms of CR and dist. For example, at the Parlier station, the reconstructed mean series improves CR from 34.9 to 56.3, improves dist by from 28.88 to 16.99, and improves R slightly from 0.84 to 0.85 when compared to Noah-MP (i.e., the best single model simulation at this station). The relatively better results of the reconstructed mean series are likely, since Noah-MP yields a slight positive bias whereas EPIC-pseudo yields a negative bias. By taking the mean of both estimates, the reconstructed estimates could yield an overall best performance in terms of all goodness-of-fit statistics across all stations.

## 4. Discussion

Soil temperature is simulated for four sites in California using different models (Noah-MP and EPIC) and evaluated against the ground-based measurement. There are many reasons contributing to the biases seen in EPIC and Noah-MP derived simulations. It is worth noting that ground-based measurements are not perfect and may contain measurement errors. Hence, we never expect model simulations fully replicate ground-based measurements. The positive bias seen in Noah-MP derived results may be caused by the scale mismatch between in-situ measurements and MERRA-2 products used to drive Noah-MP simulations. The negative bias seen in EPIC derived results may be caused by suboptimal system-defaulted parameterizations. This negative bias seen in EPIC was consistent with Doro et al. (2021) findings using system-defaulted parameters although with different study domains.

Despite the overall satisfactory performance achieved by the reconstructed mean time series, some caveats need to be mentioned. EPIC relies heavily on in-situ daily meteorological data as input into the system. When these data are missing, due to station maintenance or data inaccessibility, averaging EPIC and Noah-MP estimates may not ideal, with EPIC-derived estimates being unreliable. Therefore, future studies should employ advanced machine learning-based algorithms to assist with categorizing, understanding, and further determining which model is more suitable under different conditions.

## 5. Conclusions

Although both Noah-MP and EPIC models have been widely used around the world, to our current knowledge, a comparison or evaluation of both models’ performance side by side has not been done yet. The results suggest that relying only on one set of model estimates may not be optimal. By combining different model simulations (i.e., in this case, we take the mean of Noah-MP and EPIC-pseudo to reconstruct a new time series), we could maximize the benefits of each simulation and obtain more accurate soil temperature estimates. The reconstructed mean estimates can better capture soil temperature variations under different soil characteristics and different geographical conditions.

Specifically, key findings from this work include:

(1) Noah-MP shows relatively stable performance in terms of R when compared with all EPIC derived estimates.

(2) On average, EPIC-original performs slightly better than the other two EPIC approaches in terms of CR and dist;

(3) The reconstructed mean series, obtained by taking the mean of Noah-MP and EPIC-pseudo, are shown to yield the best performance.

We plan to carry out the following studies in the future:

(1) to evaluate soil temperature estimates derived from multiple models (all with irrigation scheduling modules) during heavily irrigated seasons in Central Valley California;

(2) to develop a machine learning based selector to automatically choose the best model under different scenarios;

(3) to carry out the project globally to understand how weather pattern, vegetation cover, and location affect model estimates.

Using soil temperature as an example, this work demonstrates the benefits of fusing models of different nature to obtain more accurate and stable estimates. We believe research findings from this study will provide useful insights to farmers and agriculturists for monitoring and predicting essential hydrological variables at the local scale. More accurate estimation can be extremely beneficial for efficiently planning and managing several agricultural activities, such as sowing, adding fertilizer, irrigation, and harvest.

## Figures and Tables

**Table 1:** Overview of the reviewed sources.

References	Model	Study Area	Year	Coupled Model	Simulated Parameter	Conclusion
[40]	EPIC	Two sites in Ontario and Quebec, Canada	1987–1995	No	Crop Yield (using different Potential Evapotranspiration estimation) and Soil Temperature	Adequate estimation of yield (soybean and corn), Under-prediction of soil temperature
[37]	EPIC	Alberta, Canada	1992	No	Wind Erosion	Not accurately simulated
[39]	EPIC	Experimental Farm in Foggia at Italy	1953 to 1997	No	Yield, Water use efficiency and economic analysis (from the yield)	Reliable estimates, Good Basis for Decision Support at farm level
[41]	EPIC	Five sites in the USA and one site in Breton, Canada	1938–1998	No	Soil Organic Carbon (SOC)	EPIC accounted for 69% of the variability in yields and 91% of the variability in SOC
[28]	Noah	137 stations across the USA	1979–2009	No	Soil Temperature	Better performance in the top soil layer
[42]	Noah-MP (with organic soil parameterization (OGN) and without organic soil parameterization (CTL))	BERMS site, Canada	1998–2009	No	Average sensible and latent heat flux, Soil Temperature, and soil moisture	Improved heat flux prediction in winter, OGN simulated soil temperature and soil moisture is more consistent compared to CTL
[43]	Noah-MP-Crop and Noah-MP	Bondville and Mead sites in Tibetan plateau	2001–2006	Yes (Weather Research and Forecasting (WRF))	LAI, crop yield, Soil temperature and soil moisture	Noah -MP simulated better simulation compared to Noah-MP
[44]	Cropping Model System (CSM)- CropSyst, APSIM, DSSAT, STICS, and EPIC	Seven sites in the US Pacific Northwest (PNW)	Baseline Period: 1979 to 2010 Future projection 2000–2100	No	Impact of Climate change on winter wheat crop yield (Length of growing season, Leaf Area Index, Biomass, Transpiration, Yield and Harvest Index)	Maximum uncertainty (upto 85%) in prediction of yield is found in CSM
[38]	EPIC	Five sites in the USA	1985–2014	Yes (GILSYM)	Crop Yield, number of insects per plant and impact of insecticide application	Robust model for predicting plant-insect interactions
[45]	Noah-MP	High Mountain Asia	2007-2008	No	Snow Mass, Soil Temperature and Soil Moisture	Improved predictability in snow depth, snow water equivalent, surface temperature and soil temperature
[46]	Noah-MP	Tanggula and Beiluhe in the Qinghai-Tibet Plateau (QTP)	2010–2011	No	Snow Cover, Soil Temperature and Soil Moisture	Snow sublimation, turbulent processes, soil thermal conductivity, and soil organic matter were integrated into the model to improve the soil temperature and moisture simulations
[25]	EPIC	Twenty-four sites (France, Germany, Italy, Switzerland, Australia, USA and Canada)	1990 to 2010	No	Soil temperature (using three different approaches)	Compared to EPIC Original, EPIC Pseudo and EPIC Enhanced provided more accurate results
[34]	Geo-CropSim	Nebraska, USA	2012 and 2015	Yes (PROSAIL and PhenoCrop Algorithm coupled in EPIC)	Crop Yield and Evapotranspiration	Improved simulation compared EPIC

**Table 2:** Geographic and land use information of each study site.

Study Site	Latitude	Longitude	Slope	Aspect	Elevation (ft)	Local Crop Type
Fresno	36°49′ N	119°45′ W	0.001	5.07	339	Irrigated Pasture
Modesto	37°38′ N	121°11′ W	0.002	4.90	35	Fruit and nut orchards and nursery
Parlier	36°36′ N	119°30′ W	0.132	4.512	337	Cotton, grapes, and orchards
Corcoran	36°2′25 N	119°34′22 W	0.0008	4.0967	190	Cotton and Tomato

(Source: https://cimis.water.ca.gov/Stations.aspx accessed on 22 August 2022).

**Table 3:** Statistics computed for Fresno station across all model estimates when compared against ground-based CIMIS soil temperature measurements from 2000 through 2020. (Note: only the November to March period is considered for computation).

	CR (%)	Dist (K)	R (-)
**Noah-MP**	13.2	24.5	0.83
**EPIC-original**	14.9	26.3	0.80
**EPIC-enhanced**	1.4	44.3	0.95
**EPIC-pseudo**	3.0	33.5	0.96
**Reconstructed-mean**	18.0	18.1	0.93

**Table 4:** Same as Table 3, but for Parlier station.

	CR (%)	Dist (K)	R (-)
**Noah-MP**	34.9	28.9	0.84
**EPIC-original**	22.5	46.6	0.39
**EPIC-enhanced**	31.1	32.4	0.60
**EPIC-pseudo**	31.4	32	0.64
**Reconstructed-mean**	56.3	17	0.85

**Table 5:** Same as Table 3, but for Modesto station.

	CR (%)	Dist (K)	R (-)
**Noah-MP**	15.7	27.4	0.87
**EPIC-original**	18.5	24.4	0.84
**EPIC-enhanced**	2.4	34.5	0.93
**EPIC-pseudo**	5.6	28.5	0.91
**Reconstructed-mean**	19.1	20.8	0.93

**Table 6:** Same as Table 3, but for Corcoran station.

	CR (%)	Dist (K)	R (-)
**Noah-MP**	22.9	23.7	0.87
**EPIC-original**	12.3	31.7	0.84
**EPIC-enhanced**	0.14	69.0	0.87
**EPIC-pseudo**	8.2	34.5	0.94
**Reconstructed-mean**	20.2	23.5	0.93

## Data Availability

Not applicable.

## References

[1] Fereres E., Orgaz F., Gonzalez-Dugo V. (2011). Reflections on food security under water scarcity. J. Exp. Bot..

[2] Bell J.E., Autry C.W., Mollenkopf D.A., Thornton L.M. (2012). A Natural Resource Scarcity Typology: Theoretical Foundations and Stra-tegic Implications for Supply Chain Management: A Natural Resource Scarcity Typology. J. Bus. Logist..

[3] Jones B.T., Mattiacci E., Braumoeller B.F. (2017). Food scarcity and state vulnerability: Unpacking the link between climate variability and violent unrest. J. Peace Res..

[4] Kummu M., Guillaume J.H.A., de Moel H., Eisner S., Flörke M., Porkka M., Siebert S., Veldkamp T.I.E., Ward P.J. (2016). The world’s road to water scarcity: Shortage and stress in the 20th century and pathways towards sustainability. Sci. Rep..

[5] Marston L.T., Read Q.D., Brown S.P., Muth M.K. (2021). Reducing Water Scarcity by Reducing Food Loss and Waste. Front. Sustain. Food Syst..

[6] Amusan L., Oyewole S. (2022). Precision agriculture and the prospects of space strategy for food security in Africa. Afr. J. Sci. Technol. Innov. Dev..

[7] Kirkaya A. (2020). Smart farming- precision agriculture technologies and practices. JSP.

[8] Trivelli L., Apicella A., Chiarello F., Rana R., Fantoni G., Tarabella A. (2019). From precision agriculture to Industry 4.0: Unveiling technological connections in the agrifood sector. BFJ.

[9] Kehl J. (2020). Moving beyond the Mirage: Water Scarcity and Agricultural Use Inefficiency in USA. Water.

[10] Thenkabail P.S. (2010). Global Croplands and their Importance for Water and Food Security in the Twenty-first Century: Towards an Ever Green Revolution that Combines a Second Green Revolution with a Blue Revolution. Remote Sens..

[11] Shu L., Hancke G.P., Abu-Mahfouz A.M. (2021). Guest Editorial: Sustainable and Intelligent Precision Agriculture. IEEE Trans. Ind. Inform..

[12] Lee C.L., Strong R., Dooley K.E. (2021). Analyzing Precision Agriculture Adoption across the Globe: A Systematic Review of Scholar-ship from 1999–2020. Sustainability.

[13] Linaza M.T., Posada J., Bund J., Eisert P., Quartulli M., Döllner J., Pagani A., Olaizola I.G., Barriguinha A., Moysiadis T. (2021). Data-Driven Artificial Intelligence Applications for Sus-tainable Precision Agriculture. Agronomy.

[14] Mafuta M., Zennaro M., Bagula A., Ault G., Gombachika H., Chadza T. Successful deployment of a Wireless Sensor Network for precision agriculture in Malawi. Proceedings of the IEEE 3rd International Conference on Networked Embedded Systems for Every Application (NESEA).

[15] Miles C. (2019). The combine will tell the truth: On precision agriculture and algorithmic rationality. Big Data Soc..

[16] Awasthi A., Reddy S.R.N. (2013). Monitoring for Precision Agriculture using Wireless Sensor Network—A Review. Glob. J. Comput. Sci. Technol..

[17] (2015). Precision Agriculture: Tomorrow’s Technology for Today’s Farmer. J. Food Process. Technol..

[18] Shafi U., Mumtaz R., García-Nieto J., Hassan S.A., Zaidi S.A.R., Iqbal N. (2019). Precision Agriculture Techniques and Practices: From Considerations to Applications. Sensors.

[19] Onwuka B. (2018). Effects of Soil Temperature on Some Soil Properties and Plant Growth. APAR.

[20] Schoonover J.E., Crim J.F. (2015). An Introduction to Soil Concepts and the Role of Soils in Watershed Management. J. Contemp. Water Res. Educ..

[21] Dong X., Xu W., Zhang Y., Leskovar D.I. (2016). Effect of Irrigation Timing on Root Zone Soil Temperature, Root Growth and Grain Yield and Chemical Composition in Corn. Agronomy.

[22] Adak T., Kumar G., Sharma P.K., Srivastava A.K., Chakravarty N. (2011). Seasonal changes in soil temperature within mustard crop stand. J. Agrometeorol..

[23] Oliveira J., Timm L., Tominaga T., Cássaro F., Reichardt K., Bacchi O., Neto D.D., Câmara G.D.S. (2001). Soil temperature in a sugar-cane crop as a function of the management system. Plant Soil.

[24] Alvarado V., Bradford K.J. (2002). A hydrothermal time model explains the cardinal temperatures for seed germination: Hydrother-mal time model of seed germination. Plant Cell Environ..

[25] Doro L., Wang X., Ammann C., De Antoni Migliorati M., Grünwald T., Klumpp K., Loubet B., Pattey E., Wohlfahrt G., Williams J.R. (2021). Improving the simulation of soil temperature within the EPIC model. Environ. Model. Softw..

[26] Smith W.N., Grant B.B., Desjardins R.L., Rochette P., Drury C.F., Li C. (2008). Evaluation of two process-based models to estimate soil N_2_O emissions in Eastern Canada. Can. J. Soil Sci..

[27] Archontoulis S.V., Miguez F.E., Moore K.J. (2014). Evaluating APSIM Maize, Soil Water, Soil Nitrogen, Manure, and Soil Temperature Modules in the Midwestern United States. Agron. J..

[28] Xia Y., Ek M., Sheffield J., Livneh B., Huang M., Wei H., Feng S., Luo L., Meng J., Wood E. (2013). Validation of Noah-Simulated Soil Temperature in the North American Land Data Assimilation System Phase 2. J. Appl. Meteorol. Clim..

[29] Stefan V., Merlin O., Er-Raki S., Escorihuela M.J., Khabba S. (2015). Consistency between In Situ, Model-Derived and High-Resolution-Image-Based Soil Temperature Endmembers: Towards a Robust Data-Based Model for Multi-Resolution Monitoring of Crop Evap-otranspiration. Remote Sens..

[30] Arsenault R., Essou G.R.C., Brissette F.P. (2017). Improving Hydrological Model Simulations with Combined Multi-Input and Multimodel Averaging Frameworks. J. Hydrol. Eng..

[31] Arsenault R., Gatien P., Renaud B., Brissette F., Martel J.-L. (2015). A comparative analysis of 9 multi-model averaging approaches in hydrological continuous streamflow simulation. J. Hydrol..

[32] Lambert S.J., Boer G.J. (2001). CMIP1 evaluation and intercomparison of coupled climate models. Clim. Dyn..

[33] Nicolle P., Pushpalatha R., Perrin C., François D., Thiéry D., Mathevet T., Le Lay M., Besson F., Soubeyroux J.-M., Viel C. (2014). Benchmarking hydrological models for low-flow simulation and forecasting on French catchments. Hydrol. Earth Syst. Sci..

[34] Bandaru V., Yaramasu R., Jones C., César Izaurralde R., Reddy A., Sedano F., Daughtry C.S., Becker-Reshef I., Justice C. (2022). Geo-CropSim: A Geo-spatial crop simula-tion modeling framework for regional scale crop yield and water use assessment. ISPRS J. Photogramm. Remote Sens..

[35] Cabelguenne M., Jones C.A., Marty J.R., Dyke P.T., Williams J.R. (1990). Calibration and validation of EPIC for crop rotations in southern France. Agric. Syst..

[36] Kiniry J.R., Blanchet R., Williams J.R., Texier V., Jones C.A., Cabelguenne M. (1992). Sunflower simulation using the EPIC and ALMA-NAC models. Field Crops Res..

[37] Potter K.N., Williams J.R., Larney F.J., Bullock M.S. (1998). Evaluation of EPIC’s wind erosion submodel using data from southern Al-berta. Can J Soil Sci..

[38] Rasche L., Taylor R.A.J. (2019). EPIC-GILSYM: Modelling crop-pest insect interactions and management with a novel coupled crop-insect model. J. Appl. Ecol..

[39] Rinaldi M. (2001). Application of EPIC model for irrigation scheduling of sun¯ower in Southern Italy. Agric. Water Manag..

[40] Roloff G., Dejong R., Nolin M.C. (1998). Crop yield, soil temperature and sensitivity of EPIC under central-eastern Canadian condi-tions. Can. J. Soil Sci..

[41] Izaurralde R.C., Williams J.R., McGill W.B., Rosenberg N.J., Jakas M.C.Q. (2006). Simulating soil C dynamics with EPIC: Model descrip-tion and testing against long-term data. Ecol. Model..

[42] Chen L., Li Y., Chen F., Barr A., Barlage M., Wan B. (2016). The incorporation of an organic soil layer in the Noah-MP land surface modeland its evaluation over a boreal aspen forest. Atmos. Chem. Phys..

[43] Liu X., Chen F., Barlage M., Zhou G., Niyogi D. (2016). Noah-MP-Crop: Introducing dynamic crop growth in the Noah-MP land sur-face model: Noah-MP-Crop. J. Geophys. Res. Atmos..

[44] Ahmed M., Stöckle C.O., Nelson R., Higgins S. (2017). Assessment of Climate Change and Atmospheric CO2 Impact on Winter Wheat in the Pacific Northwest Using a Multimodel Ensemble. Front. Ecol. Evol..

[45] Xue Y., Houser P.R., Maggioni V., Mei Y., Kumar S.V., Yoon Y. (2019). Assimilation of Satellite-Based Snow Cover and Freeze/Thaw Observations Over High Mountain Asia. Front. Earth Sci..

[46] Li X., Wu T., Zhu X., Jiang Y., Hu G., Hao J., Ni J., Li R., Qiao Y., Yang C. (2020). Improving the Noah-MP Model for Simulating Hydrothermal Regime of the Active Layer in the Permafrost Regions of the Qinghai-Tibet Plateau. J. Geophys. Res. Atmos..

[47] Guan Y., Shen Y., Mohammadi B., Sadat M.A. (2020). Estimation of Soil Temperature Based on Meteorological Parameters by the HY-BRID INVASIVE Weed Optimization Algorithm Model. IOP Conf. Ser. Earth Environ. Sci..

[48] Jimeno-Sáez P., Senent-Aparicio J., Pérez-Sánchez J., Pulido-Velazquez D. (2018). A Comparison of SWAT and ANN Models for Dai-ly Runoff Simulation in Different Climatic Zones of Peninsular Spain. Water.

[49] Shortridge J.E., Guikema S.D., Zaitchik B.F. (2016). Machine learning methods for empirical streamflow simulation: A comparison of model accuracy, interpretability, and uncertainty in seasonal watersheds. Hydrol. Earth Syst. Sci..

[50] Tabari H., Marofi S., Ahmadi M. (2010). Long-term variations of water quality parameters in the Maroon River, Iran. Environ. Monit. Assess..

[51] Diffenbaugh N.S., Swain D.L., Touma D. (2015). Anthropogenic warming has increased drought risk in California. Proc. Natl. Acad. Sci. USA.

[52] Luo L., Apps D., Arcand S., Xu H., Pan M., Hoerling M. (2017). Contribution of temperature and precipitation anomalies to the Cali-fornia drought during 2012-2015: Contribution of T and P to CA drought. Geophys. Res. Lett..

[53] Mann M.E., Gleick P.H. (2015). Climate change and California drought in the 21st century. Proc. Natl. Acad. Sci. USA.

[54] Reiter M.E., Elliott N.K., Jongsomjit D., Golet G.H., Reynolds M.D. (2018). Impact of extreme drought and incentive programs on flood-ed agriculture and wetlands in California’s Central Valley. PeerJ.

[55] Robeson S.M. (2015). Revisiting the recent California drought as an extreme value. Geophys. Res. Lett..

[56] Williams A.P., Seager R., Abatzoglou J.T., Cook B.I., Smerdon J.E., Cook E.R. (2015). Contribution of anthropogenic warming to Califor-nia drought during 2012–2014. Geophys. Res. Lett..

[57] Kumar S., Peters-Lidard C., Tian Y., Houser P., Geiger J., Olden S., Lighty L., Eastman J., Doty B., Dirmeyer P. (2006). Land information system: An interoperable framework for high resolution land surface modeling. Environ. Model. Softw..

[58] Reichle R.H., Liu Q., Koster R.D., Draper C.S., Mahanama S.P.P., Partyka G.S. (2017). Land Surface Precipitation in MERRA-2. J. Clim..

[59] Cosgrove B.A., Lohmann D., Mitchell K.E., Houser P.R., Wood E.F., Schaake J.C., Robock A., Marshall C., Sheffield J., Duan Q.Y. (2003). Real-time and retrospective forcing in the North American Land Data Assimilation System (NLDAS) project. J. Geophys. Res. Atmos..

[60] Case J.L., Crosson W.L., Kumar S.V., Lapenta W.M., Peters-Lidard C.D. (2008). Impacts of High-Resolution Land Surface Initialization on Regional Sensible Weather Forecasts from the WRF Model. J. Hydrometeorol..

[61] Niu G.Y., Yang Z.L., Mitchell K.E., Chen F., Ek M.B., Barlage M., Kumar A., Manning K., Niyogi D., Rosero E. (2011). The community Noah land surface model with multipa-rameterization options (Noah-MP): 1. Model description and evaluation with local-scale measurements. J. Geophys. Res..

[62] Putman J., Williams J., Sawyer D. (1988). Using the erosion-productivity impact calculator (EPIC) model to estimate the impact of soil erosion for the 1985 RCA appraisal. J. Soil Water Conserv..

[63] Brown R.A., Rosenberg N.J. (1999). Climate Change Impacts on the Potential Productivity of Corn and Winter Wheat in Their Primary United States Growing Regions. Clim. Chang..

[64] Taylor J.R. (1997). An Introduction to Error Analysis: The Study of Uncertainties in Physical Measurements.

